# Vaccine Immunity Against Pneumococcus in Children With Cochlear Implants

**DOI:** 10.1097/INF.0000000000004999

**Published:** 2025-09-26

**Authors:** Audrey Hominal, Renato Gualtieri, Barbara Lemaitre, Klara M. Pósfay-Barbe, Helene Cao-Van, Geraldine Blanchard-Rohner

**Affiliations:** From the * Faculty of Medicine, Geneva University; † Division of General Pediatrics, Department of Pediatrics, Gynecology and Obstetrics, Geneva University Hospitals, Geneva; ‡ Pediatrics Unit, Department of Women-Mother-Child, Lausanne University Hospital, University of Lausanne, Lausanne; § Vaccinology Laboratory; ¶ Pediatric Otolaryngology Unit, Department of Otorhinolaryngology, Head and Neck Surgery; ∥ Unit of Immunology, Vaccinology, and Rheumatology, Division of General Pediatrics, Department of Pediatrics Gynecology and Obstetrics, Geneva University Hospitals, Geneva, Switzerland.

**Keywords:** cochlear implants, pneumococcus, vaccines, serology

## Abstract

**Background:**

This study aims to assess whether Swiss guidelines for pneumococcal vaccination in children with cochlear implants are followed and whether they elicit adequate pneumococcal vaccine immunity.

**Methods:**

We performed a retrospective analysis at the Western Switzerland University Cochlear Implants Center, reviewing data between January 2009 and December 2023. Vaccination records and serotype-specific pneumococcal IgG concentrations were extracted from computerized medical records.

**Results:**

Fifty children were included, with a median implantation age of 1.5 years. In children <2 years old, 82% (27/33) were up to date with routine pneumococcal vaccination (3 doses of the 13-valent pneumococcal conjugate vaccine administered at 2, 4 and 12 months), yet only 56% (15/27) achieved protective pneumococcal seroprotection. In contrast, among children ≥2 years of age, 24% (4/17) received both the age-appropriate routine schedule and the additional recommended dose of 13-valent pneumococcal conjugate before implantation, and all of these (100%) showed protective seroprotection. An overall decline in seroprotection was observed within 5 years postvaccination, particularly around 5 years of age. Vaccine-induced immunity differed by serotype; serotypes 6B, 14 and 19 elicited higher antibody levels, whereas serotypes 4, 9V and 18C produced lower responses. Notably, children 2–5 years of age tended to exhibit lower overall pneumococcal immunity.

**Conclusions:**

Our findings support the proactive administration of an additional pneumococcal vaccine dose at the time of planning cochlear implant surgery for children ≥2 years old. In addition, periodic monitoring of serotype-specific pneumococcal antibody levels (every 5 years) is recommended to determine the need for booster vaccinations.

## Cochlear Implants in Children With Deafness: Benefits and Risks

Children with deafness may benefit from cochlear implants (CIs) when conventional hearing aids are no longer effective. Those born with profound deafness can be eligible for CI treatment as early as 10 months of age. When vestibular function is preserved, implantation is typically performed sequentially, with at least a 3-month interval between surgeries on each ear. However, if vestibular function is absent in both ears, simultaneous bilateral implantation is preferred.

Children with CI are at a higher risk of developing bacterial meningitis and acute otitis media compared with their age-matched peers.^1,2^ In 2002, US data indicated that children under 6 with CIs were up to 30 times more likely to develop pneumococcal meningitis.^3^ More recent evidence, however, suggests a substantial reduction in this risk. A 2022 meta-analysis reported a very low incidence (0.02%) of meningitis among children with inner ear malformations and CIs.^4^ This decline is largely attributed to the widespread implementation of pneumococcal vaccination programs and the withdrawal in 2002 of a specific CI device that included an intracochlear positioner—a known risk factor for meningitis.^4^

In Switzerland, the incidence of *Streptococcus pneumoniae* meningitis among CI patients remains unknown, as such data are not recorded in the “CICH database—Swiss Cochlear Implant Database, Swiss Platform for Medical Registers.”^5^

*S. pneumoniae* is a bacterium characterized by a polysaccharide capsule, which defines its more than 90 known serotypes—though only some are associated with invasive disease.^6^ Colonization of the nasopharynx, particularly in childhood, serves as its natural reservoir.^6^ The bacterium is transmitted via respiratory droplets or by contiguous spread,^6,7^ and it can cause a range of illnesses. These include noninvasive diseases, such as acute otitis media, sinusitis and pneumonia, as well as invasive pneumococcal diseases, such as complicated pneumonia, bacteremia, meningitis and arthritis, all of which can be life-threatening.^6,7^

Since the introduction of pneumococcal conjugate vaccines (PCVs) for children under 5 years of age, the incidence of invasive pneumococcal diseases in Switzerland has significantly decreased.^8^

## Pneumococcal Vaccination in Children With CIs

Historically, pneumococcal vaccination for children at risk began with the 23-valent pneumococcal polysaccharide vaccine (PPV23, Pneumovax 23), introduced about 40 years ago. This vaccine, which covers 23 serotypes (1, 2, 3, 4, 5, 6B, 7F, 8, 9N, 9V, 10A, 11A, 12F, 14, 15B, 17F, 18C, 19A, 19F, 20, 22F, 23F and 33F), was recommended for at-risk children over 2 years old.^9^ However, due to its limited efficacy and concerns regarding hyporesponsiveness, it is no longer recommended in Switzerland since 2014.^9,10^

In 2000, the 7-valent PCV (PCV7, Prevenar 7) was introduced for children under 2 years of age, and by 2001 it was recommended for all at-risk children under 5.^9,11^ From late 2005, it was also recommended as an additional vaccine for children under 2.^9^ At the end of 2010, the 13-valent PCV (PCV13, Prevenar 13) replaced PCV7, covering additional serotypes (1, 3, 4, 5, 6A, 6B, 7F, 9V, 14, 18C, 19A, 19F and 23F). Initially introduced as an additional vaccine, PCV13 became the primary vaccine for children under 5 between 2019 and 2024.^8,9,11^ As of 2025, recommendations have changed, and the 15-valent PCV (PCV15) is now advised for the primary vaccination series, along with an additional “higher valent” vaccine dose for at-risk groups, who were already vaccinated with the PCV13.^12^

Having a CI in place or planned is classified as a risk factor for invasive pneumococcal diseases.^6^ Therefore, physicians managing CI patients are advised to verify pneumococcal vaccination status and ensure that vaccinations follow the guidelines of the Swiss Federal Office of Public Health (FOPH) (see Table 1).^13–17^

**TABLE 1.** Vaccine Recommendations in Switzerland for Children With Cochlear Implants in 2023

Country	Vaccination	Age	Vaccine	Recommended Vaccination Schedule (mo)	Total Number of Doses
Switzerland^13^	Basic	<2 years, born at term <2 years, born prematurely (at less than 32, 0/7 weeks gestation or <1500 g at birth)	PCV13* PCV13*	2, 4, 12 2, 3, 4, 12	3 4
	CI	≥2 years	PCV13*	Primary vaccination series followed by 1 additional dose as soon as implantation is recommended or planned	4 or 5

* It should be noted that these recommendations were valid during the study. However, they are no longer valid now. It is now recommended to use PCV13 or PCV15 as primary series and 1 extra “higher-valent” than PCV13 in risk groups.^12^

As of 2023, pneumococcal vaccination in Switzerland, as in many other countries, is recommended for all children. This typically includes 3 doses in the 1st year of life and 4 doses for preterm infants (Table 1 and Table, Supplemental Digital Content 1, https://links.lww.com/INF/G372).^13–17^ In 2009, Switzerland recommended an additional PCV dose for children with CIs to reduce their increased risk of invasive pneumococcal disease, particularly meningitis, during the high-risk period around implantation.^18^ Vaccination schedules, vaccine types and dosing regimens for term-born CI patients vary across countries such as the USA, France and Australia, with some nations still recommending the polysaccharide vaccine for these patients (Table, Supplemental Digital Content 1, https://links.lww.com/INF/G372).^13–17^ Currently, no country recommends routine serologic monitoring or booster doses specifically for children with CI. Furthermore, the long-term persistence of vaccine-induced protection after a single PCV dose (eg, PCV13) remains uncertain. It is unclear whether booster doses or periodic serologic testing should be routinely advised.

To date, the monitoring of vaccination status for CI candidates has not been carried out systematically at our center. The purpose of our study was to assess adherence to current pneumococcal vaccination recommendations from the Swiss Federal Office of Public Health (FOPH) among children with CIs, to update the preoperative vaccination protocol for children who are candidates for CI. It also sought to evaluate whether vaccination results in sustained immunity, to determine whether additional vaccine doses might be necessary, and to investigate immune responses to different pneumococcal serotypes.

## MATERIALS AND METHODS

### Study Design and Setting

This retrospective study included children followed at the Western Switzerland University Cochlear Implants Center (CURIC), University Hospitals of Geneva (HUG), Switzerland. This study is monocentric. The CURIC performs an average of 65 cochlear implantations per year, of which 37% involve children or young people under the age of 20. The study protocol was approved by the Ethics Committee (CCER 2020-01537).

### Study Population and Inclusion Criteria

To be included in the study, children had to be followed at CURIC and be under 16 years of age at the time of their first CI. They must have received the implant between January 1, 2009, and December 31, 2023, either at CURIC or at another specialized facility. In addition, a complete vaccination record and a pneumococcal serology result were required for inclusion. All serological analyses had to be performed by the HUG Vaccinology Laboratory using the Multiplex method, as previously described.^19,20^

### Data Collection

For all children included in the study, information was extracted from the HUG computerized medical patient record.

### Pneumococcal Vaccination Status

After determining the age at first cochlear implantation, vaccination records were reviewed according to the Swiss vaccination recommendations: the basic vaccination schedule for children under 2 years of age at the time of implantation, and the basic schedule plus 1 additional dose for those 2 years of age or older at implantation (see Table 1).^13–17^ If the definitions were met, vaccination status was considered “up-to-date,” and if not, “not up-to-date.”

### Pneumococcal Serology

For each child, the results of serotype-specific pneumococcal IgG concentration were recorded, along with the date on which they were carried out.

### Definition of Seroprotection

The correlate of lasting protection reported by the FOPH corresponds to a pneumococcal serotype-specific IgG antibody concentration ≥1 mg/L.^13^ The World Health Organization defines a seroprotection rate by a threshold ≥0.35 𝜇g/mL.^21^ Until now, the correlate of clinical protection remains unknown. Clinically, at the HUG, due to the vulnerability of patients with CI, the seroprotection rate is defined by a pneumococcal serotype-specific IgG antibody concentration ≥0.5 mg/L and an overall seroprotection when this is achieved for ≥4/7 pneumococcal serotypes included in the PCV13. Concentrations >5.0 mg/L and <0.3 mg/L were entered in the database as 5.1 mg/L and 0.29 mg/L, respectively.

### Statistical Analysis

All statistical analyses were performed with STATA IC 17.0 (STATACorp, College Station, TX) and GraphPad Prism version 6.04 for Windows (GraphPad Software, San Diego, CA; www.graphpad.com).

Descriptive statistical analyses were performed. Quantitative data were described by the median and interquartile range, and qualitative data by frequency and percentage. The concentration of antipneumococcal IgG antibodies by serotype was represented by the geometric mean concentration with its 95% confidence interval (CI). Kaplan–Meier survival analysis was used to study the time course of pneumococcal vaccine seroprotection. The event of interest was the loss of overall seroprotection. The time elapsed between the last vaccination or age and the loss of overall seroprotection or the last follow-up was determined. The number of individuals at risk was reported at 5-year intervals.

## RESULTS

**FIGURE 1.**
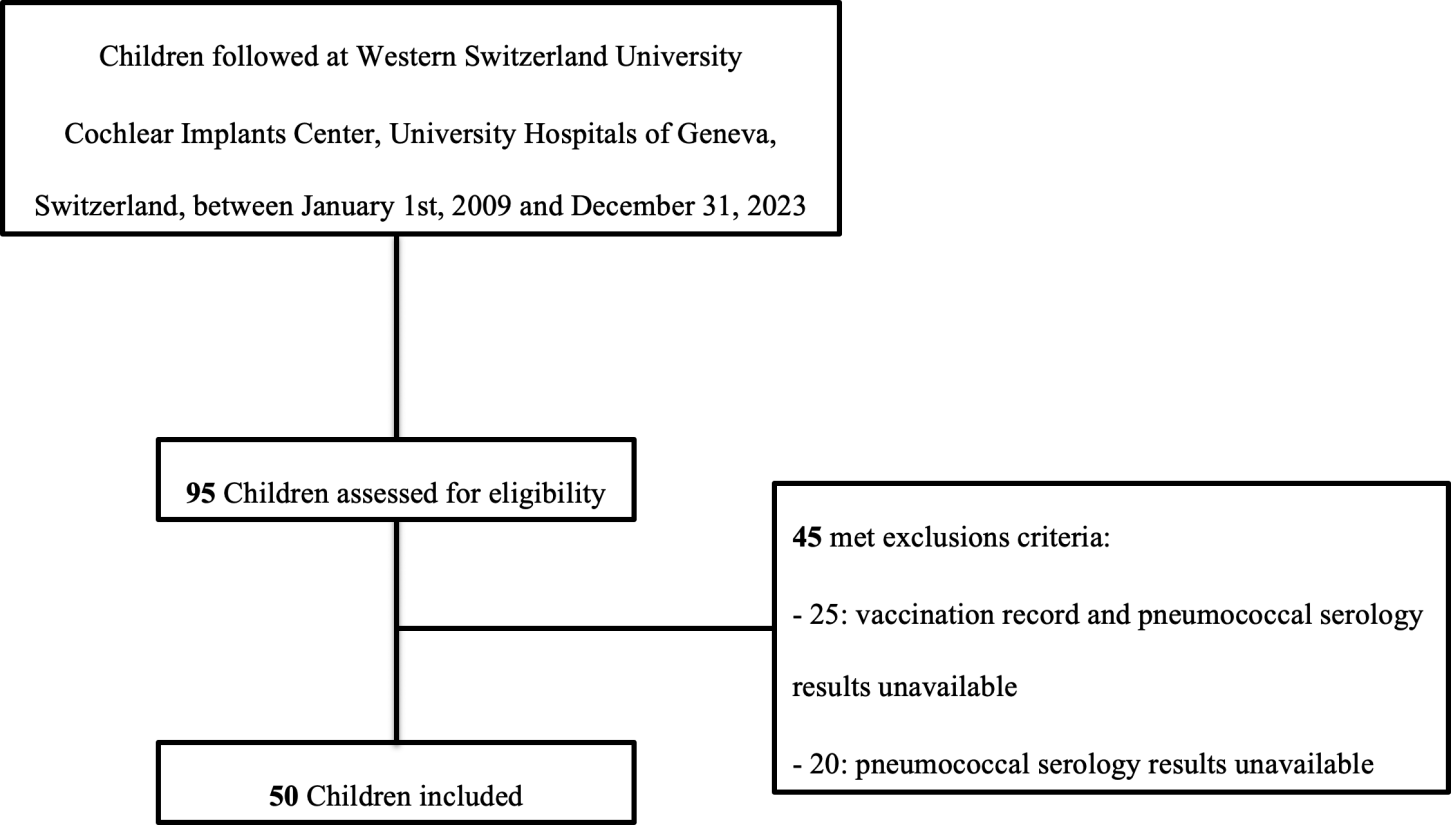
Study population flowchart.

Ninety-five CI patient medical records were reviewed. According to the study’s inclusion criteria, 50 (53%) children were included and 45 (47%) were excluded (see flowchart, Fig. 1). Results reported here focus on the children included in the study.

### Patient Characteristics

The median age of the children at implantation was 1.5 years. The characteristics of these children are detailed in Table 2. The etiology of the deafness was mainly congenital with a genetic diagnosis (50%), congenital without a genetic diagnosis (32%) or infectious (14%), but also following ototoxic medication (2%) (Figure, Supplemental Digital Content 2, https://links.lww.com/INF/G373). Among our patients, only 1 had a genetic diagnosis potentially associated with immunodeficiency (CHARGE syndrome); however, a comprehensive immunologic workup revealed normal results.

**TABLE 2.** Characteristics of the Children Included

Overall, n	50
Sex, male, n (%)	28/50 (56)
Age (yr) at implantation of 1st CI, Median (IQR)	1.5 (1.0–2.8)
Children <2 years at time of CI, n (%)	33/50 (66)
Up to date vaccination, n (%)	27/33 (82)
Seroprotected, n (%)	15/27 (56)
Not up to date, n (%)	6/33 (18)
Seroprotected, n (%)	4/6 (67)
Children ≥2 years at time of CI, n (%)	17/50 (34)
Up to date vaccination, n (%)	4/17 (24)
Seroprotected, n (%)	4/4 (100)
Not up to date, n (%)	13/17 (76)
Seroprotected, n (%)	6/13 (46)
Number of CIs	
1, n (%)	14/50 (28)
2, n (%)	36/50 (72)

Only 2 patients had a revision surgery because of local infection.

### Pneumococcal Vaccine Coverage at First CI

At the time of first CI surgery, 31 of 50 children (62%) were up-to-date for pneumococcal vaccination according to current Swiss recommendations. Among children under 2 years of age at the time of implantation, 27 of 33 (82%) had received the complete basic vaccination schedule. In contrast, among children 2 years old and older who were expected to receive an additional dose beyond the basic schedule only 4 of 17 (24%) had received this additional dose (see Table 2). All children received a PCV, either PCV13 or PCV7, depending on the year of administration.

### Pneumococcal Vaccination at First CI and Overall Seroprotection

Overall, pneumococcal vaccine seroprotection was 61% (19/31) in the 62% (31/50) of children who had an up-to-date basic vaccination when the 1st CI was implanted. More specifically, for children under 2 years of age at the time of implantation, among the 82% (27/33) of children who had received basic vaccination at the time of implantation of the 1st implant, 56% (15/27) showed a protective pneumococcal seroprotection. For the children older than 2 years at the time of implantation, among the 24% (4/17) who had received basic vaccination and an additional dose of vaccine when the 1st implant was recommended or planned, all (4/4) showed a protective pneumococcal seroprotection (see Table 2).

### Temporal Evolution of Pneumococcal Vaccine Seroprotection

**FIGURE 2.**
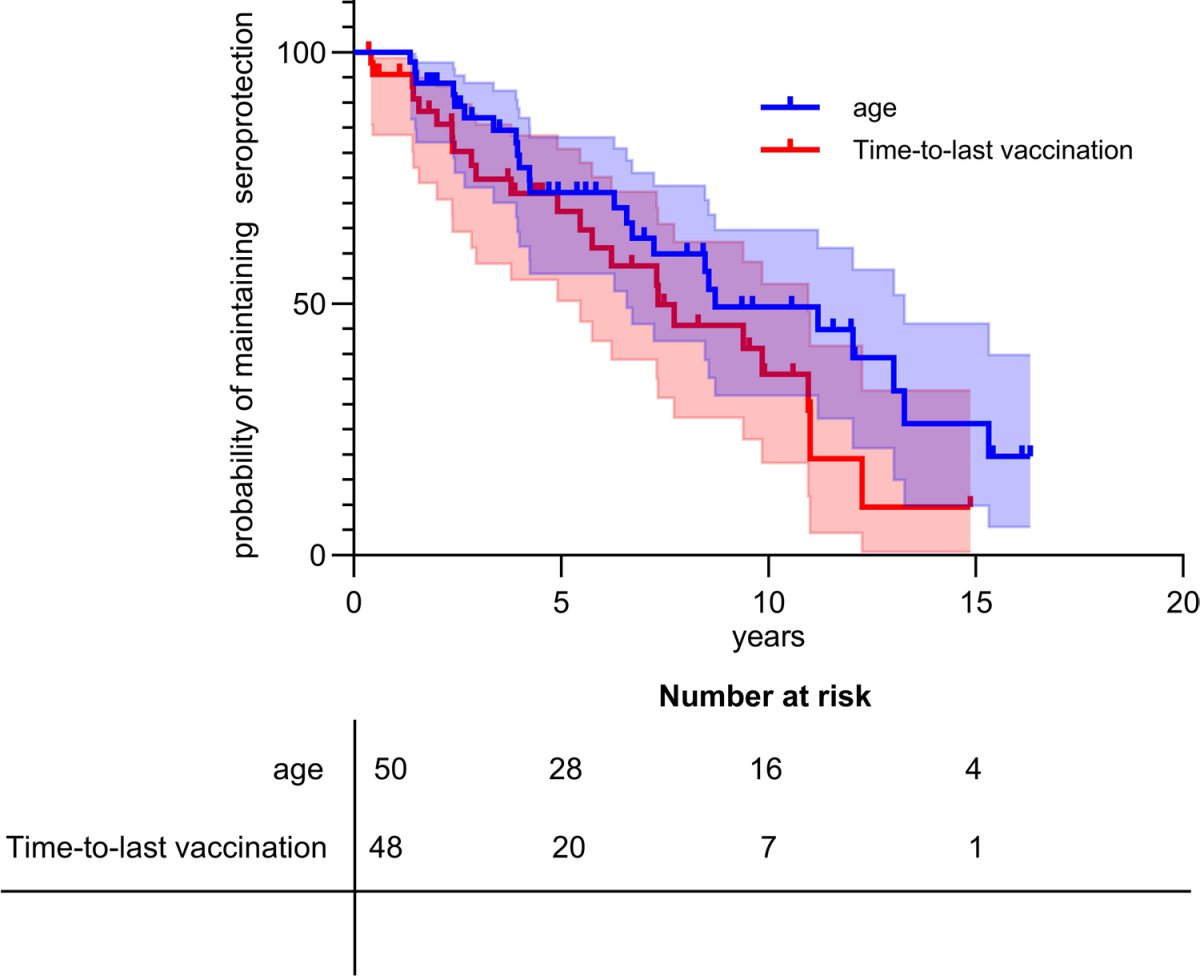
Kaplan Meier curve: probability of maintaining pneumococcal seroprotection over time. The x-axis represents the years (in blue, the age and in red, the time since last vaccination). The table below the graph represents the number of patients tested for each time points for both curves. The light blue and red zones around both curves represent the 95% CI at each time point.

To study the temporal evolution of pneumococcal seroprotection in children with CI, the time between last vaccination and loss of overall seroprotection or last follow-up was determined using Kaplan–Meier survival analyses. It showed a decrease in seroprotection during the first 5 years after vaccination and at 5 years of age (see Fig. 2). Survival analyses stratified by genetic diagnosis showed no significant differences in the probability of maintaining seroprotection over time when comparing children based on age at vaccination (log-rank test: *P* = 0.91; HR = 0.96, 95% CI: 0.41–2.24) or time since last vaccination (*P* = 0.79; HR = 1.12, 95% CI: 0.47–2.68) (Figure, Supplemental Digital Content 3, https://links.lww.com/INF/G374).

### Pneumococcal Seroprotection by Age

**FIGURE 3.**
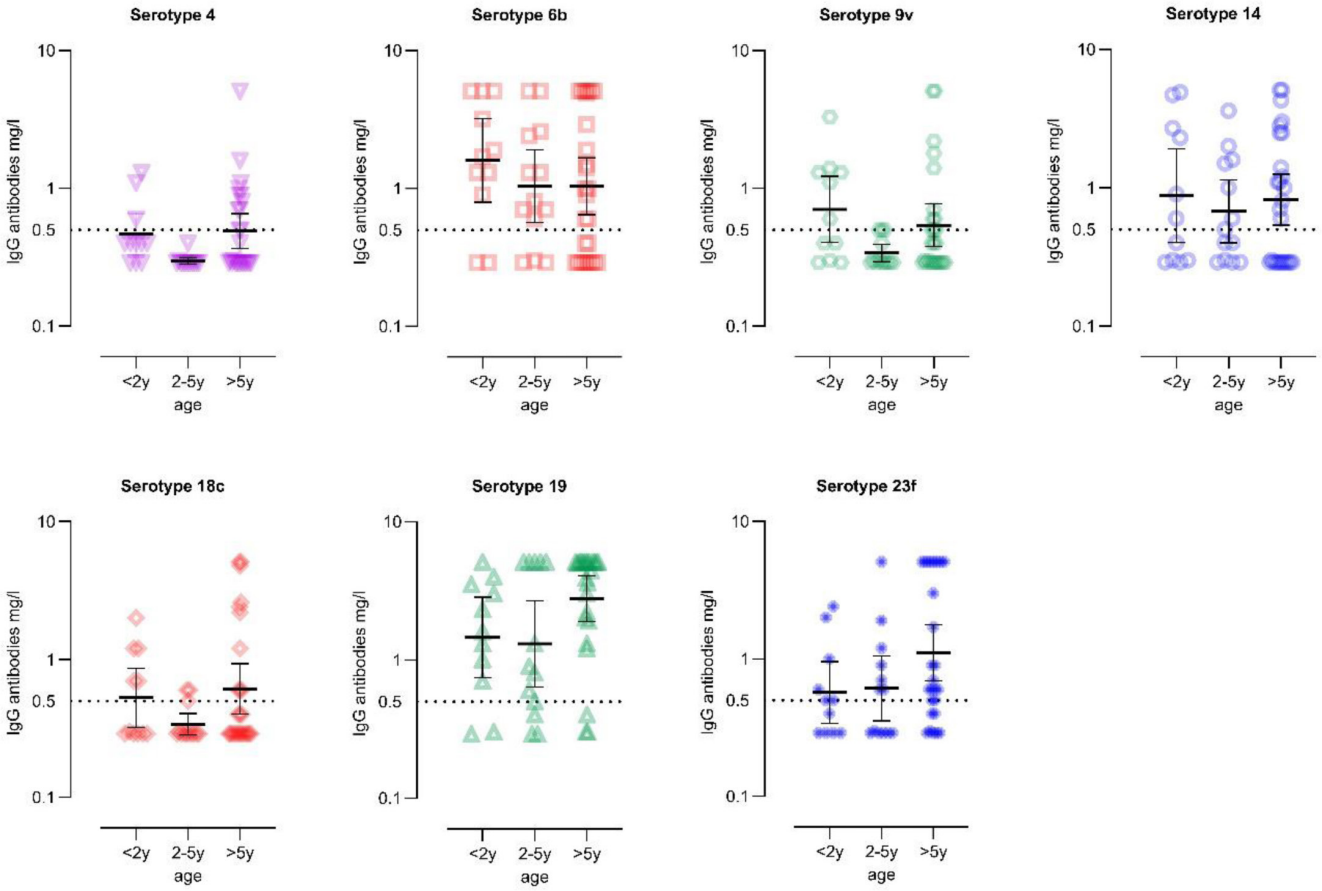
Geometric mean concentration with 95% confidence interval for 7 basic. Pneumococcal serotypes. Seroprotection rate is defined by a pneumococcal serotype-specific IgG antibody concentration ≥0.5 mg/L.

Seroprotection was higher among children over 2 years of age who were up-to-date with their vaccinations, compared with those under 2 years of age with up-to-date vaccination status (see Table 2). Vaccine-induced immunity varied across pneumococcal serotypes, with serotypes such as 6B, 14 and 19 showing higher IgG antibody levels across all age groups, whereas serotypes 4, 9V and 18C elicited lower antibody levels. Notably, children 2–5 years of age tended to exhibit lower overall pneumococcal immunity compared with other age groups (Fig. 3 and Table, Supplemental Digital Content 4, https://links.lww.com/INF/G375).

Although this study was not specifically designed to assess that outcome, we can confirm that none of the excluded infants or children in our patient group were hospitalized for otitis media or invasive pneumococcal disease during the study period.

## DISCUSSION

Our study highlights several key findings regarding pneumococcal vaccination coverage and immunity in children with CIs.

### Vaccination Documentation Challenges

Notably, 26% of children with CI followed at CURIC had no documented vaccination records. Accurate vaccination documentation is critical for establishing an appropriate immunization plan, particularly when CI implantation is considered. The lack of documentation may stem from various factors, including misplaced or forgotten records, fragmented healthcare involving multiple providers or insufficient diligence in recording vaccinations. To mitigate these issues, we advocate for the widespread adoption of electronic vaccination records, as outlined in Appendix 4 of the Swiss Confederation’s ordinance of April 2024.^22^

### Pneumococcal Vaccination Coverage and Efforts to Improve It

Our results show that pneumococcal vaccination coverage at the time of CI implantation was 82% for children under 2 years of age (basic vaccination) and only 24% for those 2 years old and older (basic vaccination plus one additional dose). The pneumococcal vaccination coverage observed in our study raises concerns, particularly given Switzerland’s well-resourced healthcare system. We believe that similar results would likely be observed in other CI centers across Switzerland, including those in the German-speaking regions, as current practices and national vaccination guidelines are applied consistently, based on discussions among physicians at national meetings. This underscores the need to harmonize clinical protocols at the national level.

Previous studies reported lower vaccination coverage, with 62% for PCV13 and 41% for PPV23 before the implementation of targeted programs aimed at improving coverage of the PPV23 dose.^23^ This is concerning and suggests that vaccination coverage in less-affluent countries, where healthcare access and vaccine monitoring systems may be more limited, could be even lower. Vaccination strategies vary across centers. They include measures such as the creation of vaccine-monitoring databases with reminder systems; requirement for written vaccination status confirmation before surgery; adherence to the UK Department of Health’s guidelines following safety alerts about increased meningitis risk among CI recipients or implementation of specific protocols to verify vaccination and actively inform parents and physicians about the importance of pneumococcal vaccination.^23–28^

Despite CURIC’s proactive approach—including a vaccination and serologic monitoring database, informational letters to families, review of vaccination and serology records and support from pediatric immuno-vaccinology specialists—vaccination rates remain suboptimal. To further enhance coverage, additional steps could include requiring written proof of vaccination before surgery and offering personalized counseling to address potential parental vaccine hesitancy.

### Vaccine-Induced Immunity and Seroprotection

To our knowledge, this is the first study to evaluate vaccine-induced immunity specifically in children with CI. Among children under 2 years of age, we found no clear association between vaccination status and overall seroprotection. However, in children 2 years old and older, all those with up-to-date vaccination demonstrated sufficient seroprotection, compared with only 46% among those who were not fully vaccinated. It should be noted that while specific data on the protective immunity status of children in Switzerland is limited, there is a high vaccination coverage, suggesting a robust level of population immunity against pneumococcal disease.^29^

Importantly, our findings also reveal a gradual decline in pneumococcal seroprotection within the first 5 years after vaccination, with some children losing protective antibody levels as early as 2 years postimmunization. These results support the need for an additional pneumococcal vaccine dose at around 2 years of age for at-risk children. Furthermore, they suggest that periodic serologic monitoring—approximately every 3 to 5 years—may be beneficial, with booster doses administered according to individual serology results.

Nonetheless, additional prospective studies are needed to determine whether the risk of pneumococcal meningitis persists over time in CI recipients, as this remains a critical question for long-term patient management.

Our study has several limitations. First, it is a retrospective analysis with a relatively small sample size and notable heterogeneity within the study population, limiting the generalizability of the findings. Moreover, although we included all children from French-speaking regions of Switzerland who underwent CI surgery in Geneva, we were unable to analyze seroprotection rates by finer age subgroups due to sample size constraints. In addition, clinical data on invasive and noninvasive pneumococcal infections were not collected, preventing us from correlating serologic findings with clinical outcomes.

Another important challenge lies in the absence of a universally accepted correlate of protection for pneumococcal disease. Currently, different thresholds are used: ≥0.5 mg/L at our institution (HUG), ≥1 mg/L as recommended by the Swiss Federal Office of Public Health (FOPH) for long-term protection and ≥0.35 μg/mL according to World Health Organization guidelines—complicating comparisons across studies.^13,21^ Despite these limitations, we believe that our findings contribute meaningfully to the growing discussion on individualized vaccination strategies for high-risk populations such as CI recipients.

### Recommendations and Future Directions

Prospective studies with larger cohorts are needed to better assess pneumococcal vaccination coverage, identify barriers to vaccination, and evaluate long-term vaccine-induced immunity in children with CI. Moreover, further research should focus on optimizing vaccination schedules and monitoring strategies to sustain adequate protection against pneumococcal disease.

## CONCLUSIONS

Clinicians managing children with CI must ensure that pneumococcal vaccination is up to date, ideally before implantation. We recommend the systematic administration of an additional pneumococcal vaccine dose as soon as CI surgery is planned. Furthermore, periodic pneumococcal serology testing, approximately every 5 years, should be considered to evaluate the need for booster doses and maintain long-term protection.

## ACKNOWLEDGMENTS

*The authors thank the Pediatric, Gynecology, and Obstetrics research platform of the Geneva University Hospital and the Western Switzerland University Cochlear Implants Center for their collaboration and support. The authors thank Ana Bel Nguyen Folgar Torres for her support with administrative assistance.*

## Supplementary Material


