# Supplementary material for: Vaccine Immunity Against Pneumococcus in Children With Cochlear Implants
Source: Pediatr Infect Dis J. 2025 Sep 26;45(2):187–93. doi: 10.1097/INF.0000000000004999 (PMC12771958; doi:10.1097/INF.0000000000004999)
Supplement: Supplementary file 1 [file inf-45-187-s001.pdf]

SUPPLEMENTAL DIGITAL CONTENT 1. Vaccine recommendations in other countries for children with cochlear implants in 2023

Country	Vaccination	Age	Vaccine	Recommended vaccination schedule (month)	Total number of doses
**USA**[14, 15]	**Basic**	All children	PCV 13/15/20	2, 4, 6, 12-15	4
	**Cochlear implant**	≥ 2 years - basic vaccination with PCV13/15	PPV23<br>Or<br>PCV20	Primary vaccination series followed by one additional dose of PPV23 at least 8 weeks after the last dose of PCV<br><br>Primary vaccination series followed by one additional dose of PCV20	5<br><br>5
		If at least one dose of PCV20 is used for basic vaccination, no additional vaccination is indicated			4
**France**[16]	**Basic**	< 2 years, born at term	PCV 13	2, 4, 11	3
	**Cochlear implant**	< 2 years or/and born prematurely<br>2-5 years	PCV13<br>PPV23	2, 3, 4, 11	4<br>4

		> 5 years	PPV23	Primary vaccination series followed by one additional dose of PPV23 at 24 months<br>After vaccination with the PCV13-PPV23 sequence, one additional dose of PPV23 at an interval of 5 years since the last dose of PPV23	/
**Australia[17]**	**Basic**	All children	PCV13	2, 4, 12	3
	**Cochlear implant**	< 12 month or/and born prematurely	PCV 13 and PPV23	PCV13 at 2, 4, 6, 12 then one dose of PPV23 at 4 years and one dose at an interval of 5 years since the last dose of PPV23	6
		≥ 12 month	PCV13 and PPV23	One dose of PCV13 at least two months after the last injection of PCV13. One year after PCV13, one dose of PPV23 followed by a	/

				second dose of PPV23 at interval of 5 years after the first	
