# Supplementary material for: Vaccine Immunity Against Pneumococcus in Children With Cochlear Implants
Source: Pediatr Infect Dis J. 2025 Sep 26;45(2):187–93. doi: 10.1097/INF.0000000000004999 (PMC12771958; doi:10.1097/INF.0000000000004999)
Supplement: Supplementary file 2 [file inf-45-187-s002.pdf]

SUPPLEMENTAL DIGITAL CONTENT 2. Causes of hearing loss in our study cohort

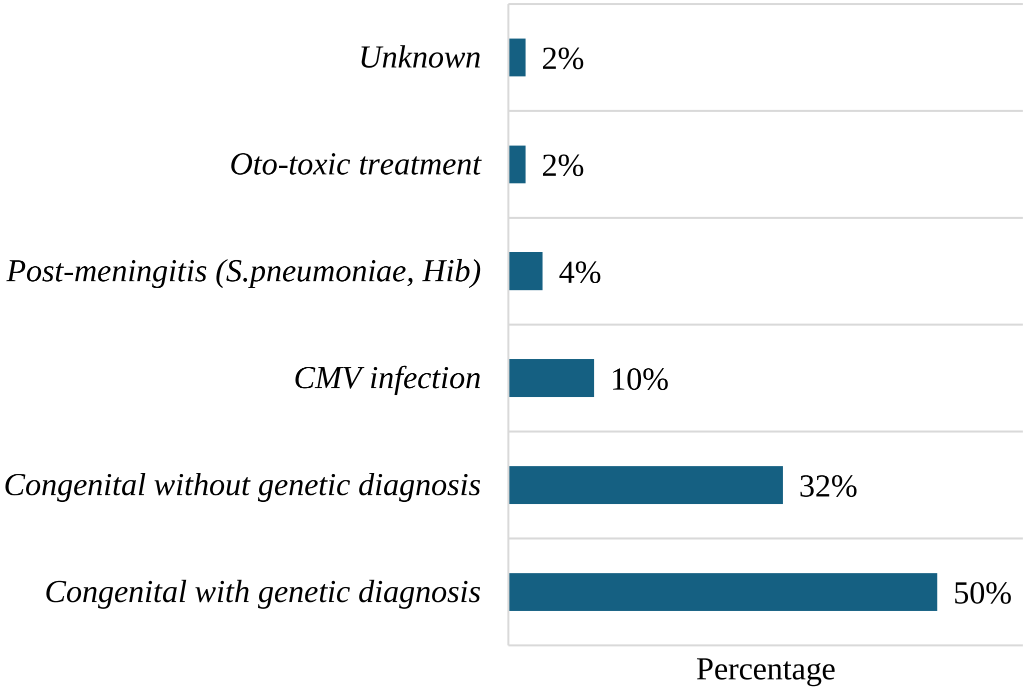

Definition:
Hib: *Haemophilus influenzae type b*
CMV: *Cytomegalovirus*
