# Supplementary material for: Vaccine Immunity Against Pneumococcus in Children With Cochlear Implants
Source: Pediatr Infect Dis J. 2025 Sep 26;45(2):187–93. doi: 10.1097/INF.0000000000004999 (PMC12771958; doi:10.1097/INF.0000000000004999)
Supplement: Supplementary file 3 [file inf-45-187-s003.pdf]

SUPPLEMENTAL DIGITAL CONTENT 3. Survival analyses stratified by genetic diagnosis.

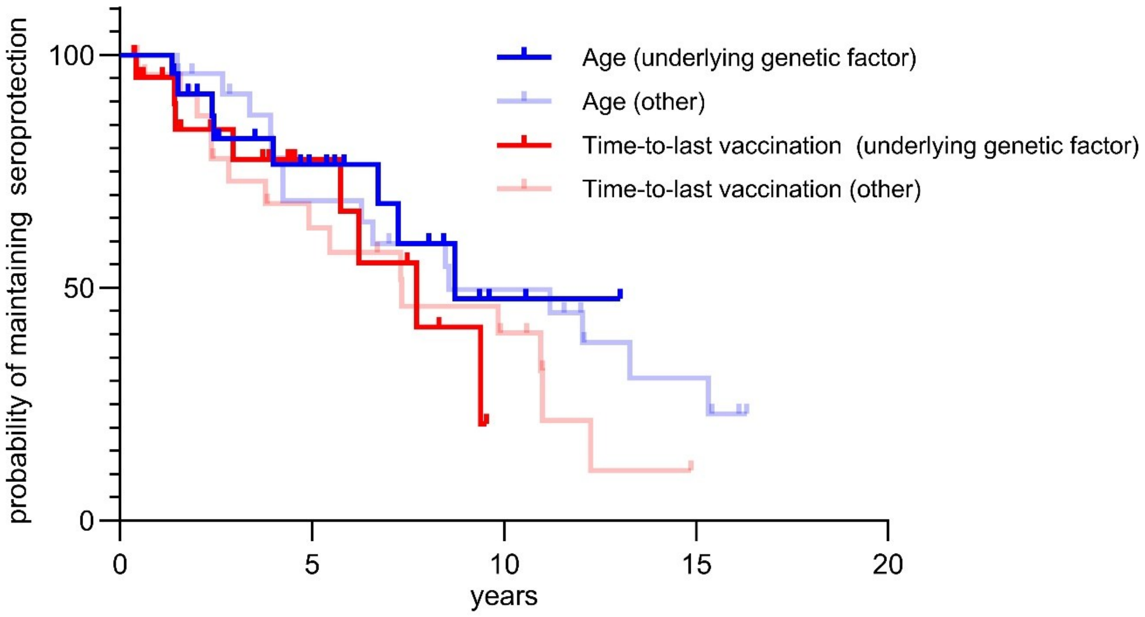

Legend:

Kaplan Meier curve: probability of maintaining pneumococcal seroprotection over time. The X axis represent the years (in blue the age and in red the time since last vaccination). The light blue and red zone around both curves represent the 95% CI at each time-point.
