# Supplementary material for: Vaccine Immunity Against Pneumococcus in Children With Cochlear Implants
Source: Pediatr Infect Dis J. 2025 Sep 26;45(2):187–93. doi: 10.1097/INF.0000000000004999 (PMC12771958; doi:10.1097/INF.0000000000004999)
Supplement: Supplementary file 4 [file inf-45-187-s004.pdf]

SUPPLEMENTAL DIGITAL CONTENT 4. Geometric mean concentration with 95% confidence interval for seven basic Pneumococcal serotypes

Age (years)	< 2	2-5	> 5
**N = 50**	**N = 11**	**N = 13**	**N = 26**
**Serotype**	**GMC [CI 95%]**	**GMC [CI 95%]**	**GMC [CI 95%]**
**4**	0.46 [0.32-0.65]	0.29 [0.28-0.31]	0.49 [0.36 -0.65]
**6B**	1.6 [0.79-3.21]	1.0 [0.56-1.9]	1.03 [0.64-1.67]
**9V**	0.70 [0.40-1.22]	0.33 [0.29-0.39]	0.54 [0.37-0.77]
**14**	0.87 [0.40-1.91]	0.67 [0.40-1.14]	0.81 [0.53-1.24]
**18C**	0.52 [0.32-0.86]	0.33 [0.28-0.41]	0.61 [0.41-0.93]
**19**	1.45 [0.74-2.84]	1.31 [0.63-2.69]	2.78 [1.91-4.07]
**23F**	0.56 [0.33-0.95]	0.61 [0.35-1.05]	1.11 [0.69-1.77]
